# Researchers’ Individual Publication Rate Has Not Increased in a Century

**DOI:** 10.1371/journal.pone.0149504

**Published:** 2016-03-09

**Authors:** Daniele Fanelli, Vincent Larivière

**Affiliations:** 1 Meta-Research Innovation Center at Stanford (METRICS), 1070 Arastradero road, Stanford University, Palo Alto, 94304, California, United States of America; 2 École de bibliothéconomie et des sciences de l'information, Université de Montréal C.P. 6128, Succ. Centre-Ville, Montréal, QC, H3C 3J7 Canada and OST-CIRST, Université du Québec à Montréal, C.P. 8888, Succ. Centre-Ville, Montréal, QC, H3C 3P8, Canada; Universidad de Las Palmas de Gran Canaria, SPAIN

## Abstract

Debates over the pros and cons of a “publish or perish” philosophy have inflamed academia for at least half a century. Growing concerns, in particular, are expressed for policies that reward “quantity” at the expense of “quality,” because these might prompt scientists to unduly multiply their publications by fractioning (“salami slicing”), duplicating, rushing, simplifying, or even fabricating their results. To assess the reasonableness of these concerns, we analyzed publication patterns of over 40,000 researchers that, between the years 1900 and 2013, have published two or more papers within 15 years, in any of the disciplines covered by the Web of Science. The total number of papers published by researchers during their early career period (first fifteen years) has increased in recent decades, but so has their average number of co-authors. If we take the latter factor into account, by measuring productivity fractionally or by only counting papers published as first author, we observe no increase in productivity throughout the century. Even after the 1980s, adjusted productivity has not increased for most disciplines and countries. These results are robust to methodological choices and are actually conservative with respect to the hypothesis that publication rates are growing. Therefore, the widespread belief that pressures to publish are causing the scientific literature to be flooded with salami-sliced, trivial, incomplete, duplicated, plagiarized and false results is likely to be incorrect or at least exaggerated.

## Introduction

Ever since the early 20th century, academic lives and careers have been guided, first in the United States and later in other countries, by a “publish or perish” philosophy whose effects are increasingly controversial [1]. Already in the 1950s, academics were publically disputing that, whilst publishing promptly one’s results is a duty for all researchers, setting explicit productivity expectations was a recipe for disaster [2]. The debate escalated after the 1980s, with the increasing adoption of formal performance evaluation practices and the growing use in such context of quantitative metrics of productivity and citation impact, such as the Impact Factor and the h-index [3–5].

Today, whenever problems of contemporary science are discussed, it is commonplace to suggest that scientists, being pressured to pad their CVs with publications, might be increasingly fractioning their results (i.e. “salami slicing” data sets to the smallest publishable unit), surreptitiously re-using data in multiple publications, duplicating their papers, publishing results that are preliminary or incomplete, underemphasizing limitations, making exaggerated claims and even resorting to data fabrication, falsification and plagiarism e.g.[6–10]. Research evaluation policies in scientifically prominent countries have reacted to these concerns by de-rewarding productivity. The German Research Foundation (DFG), for example, has imposed a limit on the number of papers that researchers can include in their CVs in support of a grant application [11]. In The Netherlands, the national research assessment exercise has revised its policies and has dropped the “productivity” category, which counted total number of publications, from its ranking system [12].

Evidence that modern science suffers from over-productivity, however, is mostly anecdotal or indirect. Scientific journals are said to be increasingly flooded with submissions, but such estimates are not adjusted for various confounding factors, and in particular the fact that the population of scientists is growing [13, 14]. Several surveys have probed scientists’ perceptions of pressures to publish, and found them to be high in all disciplines and particularly high in the United Kingdom and North America, but it is unclear how such perceptions reflect actual behaviour, or even if they may represent a self-fulfilling prophecy [15–17]. Meta-analyses suggest that positive outcome bias in the literature has increased in recent decades and might be higher in academically productive areas, especially in the United States [18–21], but the causes underlying these patterns remain highly speculative.

To the best of our knowledge, no study has conclusively assessed whether individual publication rates of scientists have actually increased as commonly speculated. In particular, whilst a rise in collaborations has been amply documented across the sciences e.g. [22] and whilst some evidence suggests that individual scientists are publishing more studies overall, at least in the physical sciences [23], no study has verified whether researchers are actually publishing more on an individual basis and independent of their specific collaboration patterns. This gap in the literature is not surprising, because assessing individual publication rates in the literature is technically very challenging.

By sampling researchers whose names included three initials (e.g. Vleminckx-SGE), we were able to analyse publication patterns of individual scientists that operated during the 20th century in all disciplines covered by the Web of Science database. Since individual careers vary widely in length, and since pressures to publish are supposed to be highest at the beginning of scientific careers, we limited all measurements to the first 15 years of publication activity. We will refer to this category as “early-career researchers”. Since our literature database included studies up to the year 2013, we could retrieve the publication patterns of 98 cohorts of early-career researchers, whose first year publication ranged between 1900 and 1998. Since publications are assumed to be crucial to survival in academia, we excluded all authors who had ceased publishing before the end of their early-career period, because these might not be representative of successful and/or active scientists. Moreover, to minimize name disambiguation errors (see Materials and Methods), analyses were limited to researchers working in the Unites States, Canada, Europe-15 countries, Australia and New Zealand. Our final sample thus included 41,427 individuals.

## Materials and Methods

To identify individual authors unambiguously, we retrieved from the Web of Science database (henceforth, WOS) all authors whose names included three or more initials (surname plus initials for first name and at least two middle names; for example, Vleminckx-SGE), a combination that greatly reduced author identification errors and makes our results conservative (see section Disambiguation error risk).

We retrieved from the WOS all records that had been co-authored by any one of these names. In order to identify early-career researchers, we selected researchers that had co-authored at least two papers, and whose papers spanned a period of at least 15 years, starting from the year of the first publication. We will refer to members of this subset as “early-career” authors.

The publication lists of each early-career author were analysed in order to extract the following information:

- year when first paper was published.
- total number of papers co-authored during the 14 years following the year of first paper.
- average number of co-authors in these papers.
- total number of citations accrued by these papers, counted at the time of data retrieval (i.e. December 2014).
- Average 5-year impact factor for these papers, normalized by discipline.
- Number of papers in which the name of the author is first in the co-author list.
- Most likely country of activity of the author.

Records in the WOS only started linking each author in a paper to his/her individual address in recent years. Earlier records only include a list of all addresses, as they appear in the paper. In order to match with certainty addresses to authors, therefore, we recorded the first country of affiliation listed in papers in which the researcher was first author. If more than one country was associated with this name over the publication period, country was attributed by majority rule. Authors for which no country could be inferred based on this method (in particular, because they had never published an article as first author), were placed in an “unknown” category, which in the full text figures is aggregated with the “other country” category.

### Disambiguation error risk

The likelihood that two authors share the same surname as well as the initials of first and two middle names is extremely low. In theory, the number of possible combinations could be as high as 26^8^, assuming an average surname length of five letters and 26 letters in the alphabet. In practice, however, surnames tend to be country-specific, and some countries are more likely to have two middle names as well as to have shorter surnames, leading to a higher theoretical homonymy rate, i.e. multiple researchers sharing the same surname and initials. Previous evidence suggests that error rate tends to be higher for authors from Latin-American and South-East Asian countries, and particularly from China, in which surnames are translated in an alphabet that renders them highly similar. Therefore all our main analyses were limited to authors that our algorithm (see above) attributed to countries from North America, Europe-15, Australia and New Zealand.

It is important to emphasize that disambiguation errors in our study are virtually unidirectional, and thus render our analysis very conservative. The main type of error that our sampling strategy might encounter is the merging of two distinct authors into one. This would inflate the apparent productivity associated with that name because the population of scientists has grown steadily over the century, which increases the likelihood that two or more scientists share the same name. Therefore, non-disambiguation errors are likely to have increased across our time series, leading to, if anything, a spurious increase in the apparent productivity of authors. The opposite error, in which the bibliography of a single individual is incorrectly split in two, may only occur when authors change their names or surnames over time, which is a relatively rare event. Therefore, the non-increasing trends we report for productivity are likely to be still over-estimating the true changes of individual productivity over time.

To estimate the rate of disambiguation error in our sample, we retrieved 50 names at random, and examined the coherence of their publication lists. This analysis showed that 47 researchers had no homonyms, and that the retrieved list of papers did not include any misattributed papers. Two researchers had one homonym to which one paper could be attributed, and one researcher name included papers from three distinct researchers.

### Representativeness of the sample

We probed the representativeness of our sampling strategy by conducting two tests. First, we assessed the prevalence of three-initialled names in the Web of Science. Since addresses in the Web of Science were not linked to author names until recently, we had to limit the analysis to first-author names, which could then be unambiguously associated to the first affiliation listed. Thus we looked at how the proportion of three-lettered individuals varied amongst first-author names associated with each country, from one year to the next. Both the magnitude and temporal change of this proportion varied significantly both in magnitude and temporal change across the countries included, showing either increases and decreases of this quantity over the years, depending on country (S1 Fig, numerical analyses in S1 File). These temporal trends are uncorrelated with the publication patterns found by our study, which are unidirectional instead. For example, the strongest declines in this proportion over the years were observed in Portugal and United Kingdom, i.e. in country that exhibit widely different trends in fractional and first-author publication rate (S1 File, and see Results).

Second, we assessed whether the names of the authors in our sample exhibited patterns that might suggest a biased sampling. In the supplementary text file we report the names of the 20 most productive and least productive authors in our sample, for United States and for the United Kingdom, for the years 1978, 1988, 1998. No overabundance of foreign names, or any other clear difference between any categories or years was evident. For example, South East Asian names were rare in all samples, independent of productivity level or year of sampling (S1 File). This suggests that our sampling strategy produced a representative sample of researchers operating in any given country or discipline.

### Analyses

Temporal trends for all parameters examined were analysed in a generalized linear model, in which year of researcher’s first publication was the independent variable, and quantities measured over the researcher’s publications of the subsequent 15-years constituted the dependent variables. The distribution of errors was modelled as quasi-Poisson for count data (i.e. number of papers, average number of co-authors, citations) and Gaussian for the remaining variables. These choices were made based on theoretical considerations dictated by the nature of the data, corroborated by an examination of the shape of the distribution of data after necessary transformations. There was no overlap in authorship amongst the publication profiles of the sampled authors, fully justifying an assumption of independence of errors. All analyses involving cross-century patterns fitted a cubic polynomial, whereas those limited to the post-1980 period fitted a first-degree polynomial (i.e. a univariate, linear regression). Cubic polynomials were preferred over more complex models because the scope of the analysis is primarily to illustrate long-term trends. The distribution of residuals was assessed for all models and was deemed sufficiently close to a normal distribution for the purposes of the analyses, which is merely descriptive and based on a very large sample—making our results not crucially dependent on null-hypothesis testing.

To assess whether analytical choices might have concealed a different trend from the one we report, we plotted the corresponding “raw” average values of fractional and first-author publication rate for each year (S2 Fig). Trends in means closely match those suggested by the regression models and confirm that average fractional and first-author publication rates in all disciplines cannot be said to have increased over the century.

### Database coverage of the literature

The WOS database only captures a portion of the literature, so we examined the possibility that the trends observed could be an artefact cause by decreasing coverage of key journals. On the contrary, we found that the WOS coverage has risen steadily for all disciplines, at rates that do not mirror the patterns we report (S3 Fig). If scientists were actually publishing more papers, they would be doing so in a decreasing number of journals and precisely in those not included in the database, which is very unlikely.

The lower coverage by the WOS of the older literature also implies that, if anything, our study might be underestimating the actual publication rate of older authors, making our test once again conservative with respect to the hypothesis of growing publication rates.

## Results

Our sampling procedure yielded an initial pool of 1,219,067 records of articles authored by 543,789 three-initialled names, of which 70,310 had authored at least two papers during fifteen years and 41,427 could be ascertained to have worked either in North America, Europe-15 or Australia/New Zealand. Hence, our final sample consisted of a total of 760,323 papers published by 41,427 authors form all disciplines in the Web of Science (Mathematics N = 492, Earth and Space Science N = 1,604, Physics N = 2,193, Chemistry N = 3,531, Biology N = 3,247, Biomedical Research N = 5,551, Clinical Medicine N = 17,928, Psychology N = 657, Social Sciences N = 1,103, Arts and Humanities N = 1,114, Other (i.e. Professional Fields+Health+Engineering and Technology) N = 3,524).

**Fig 1:**
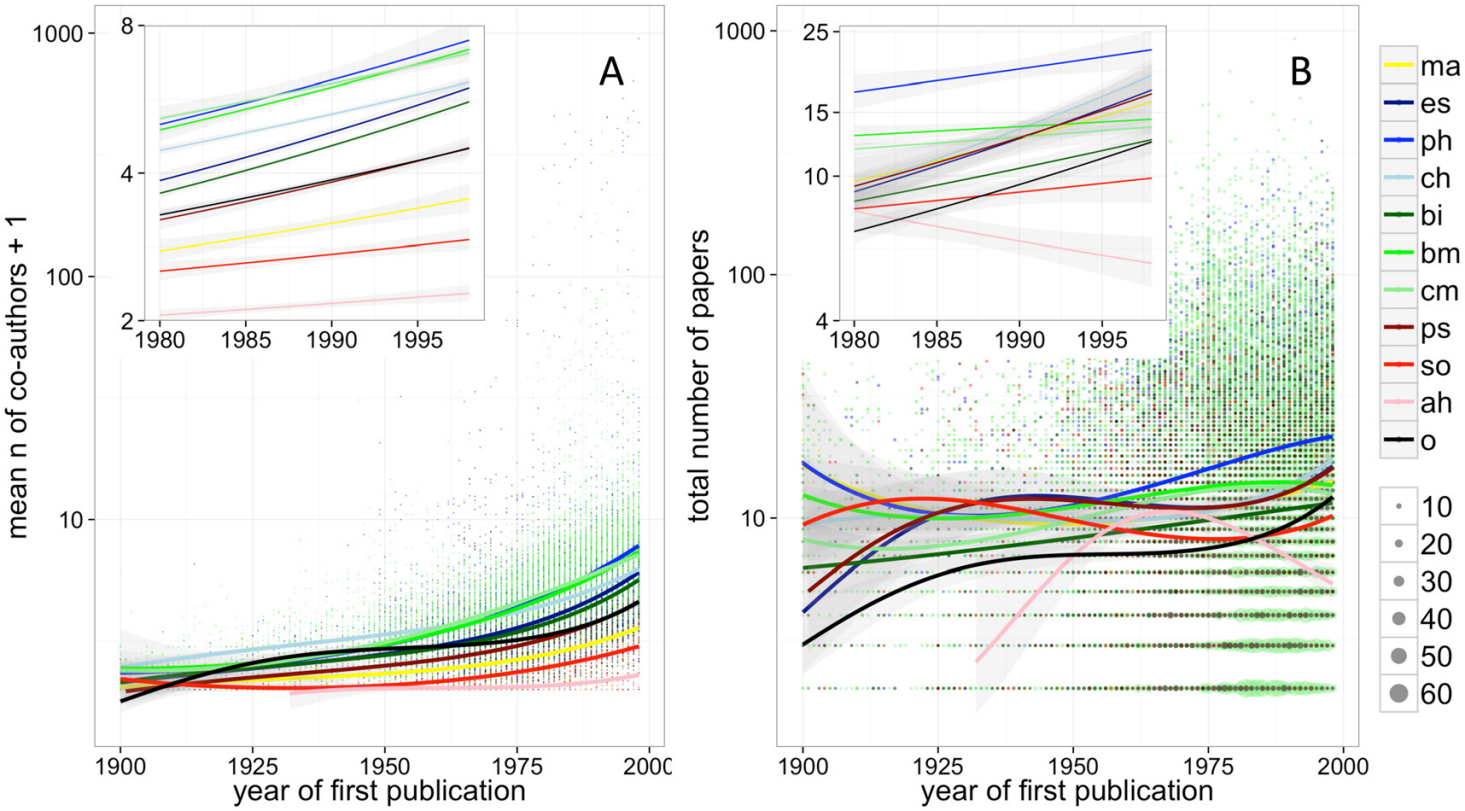
A) Average number of co-authors per paper published by individual scientists during the first 15 years of publication activity, plotted by the year of their first publication. B) Total number of papers published by individual scientists during the first 15 years of publication activity, plotted by the year of their first publication (dot size is proportional to number of overlapping data points). Boxes in both figures illustrate trends observed since the 1980s. Lines and confidence intervals are derived from a generalized linear model assuming quasi-Poisson distribution of errors (see Methods for further details). Legend: ma = mathematics, es = earth & space science, ph = physics, ch = chemistry, bi = biology, bm = biomedical research, cm = clinical medicine, ps = psychology, so = social sciences, ah = arts & humanities, o = other.

The average number of papers published by early-career researchers has been stable or increasing for all disciplines during 20^th^ century, and has increased for most disciplines after the year 1980. The number of co-authors appearing on these papers has also increased, and at a visibly faster rate than the number of publications. Scientists in all disciplines went from having almost no co-authors at the start of the century to having, by the end of it, on average between 2 and 7 in all disciplines except the Arts & Humanities ((Fig 1a and 1b; numerical results for this and all subsequent figures and analyses mentioned in the text are reported in S1 File).

Scientists’ number of collaborators significantly affected their productivity and impact. The relationship is non-linear, but the “optimal” average number of collaborators is non-zero in all disciplines, and grows along a gradient of “hardness” of subject matter [24], i.e. from the arts and humanities to the physical sciences (S4 Fig).

**Fig 2:**
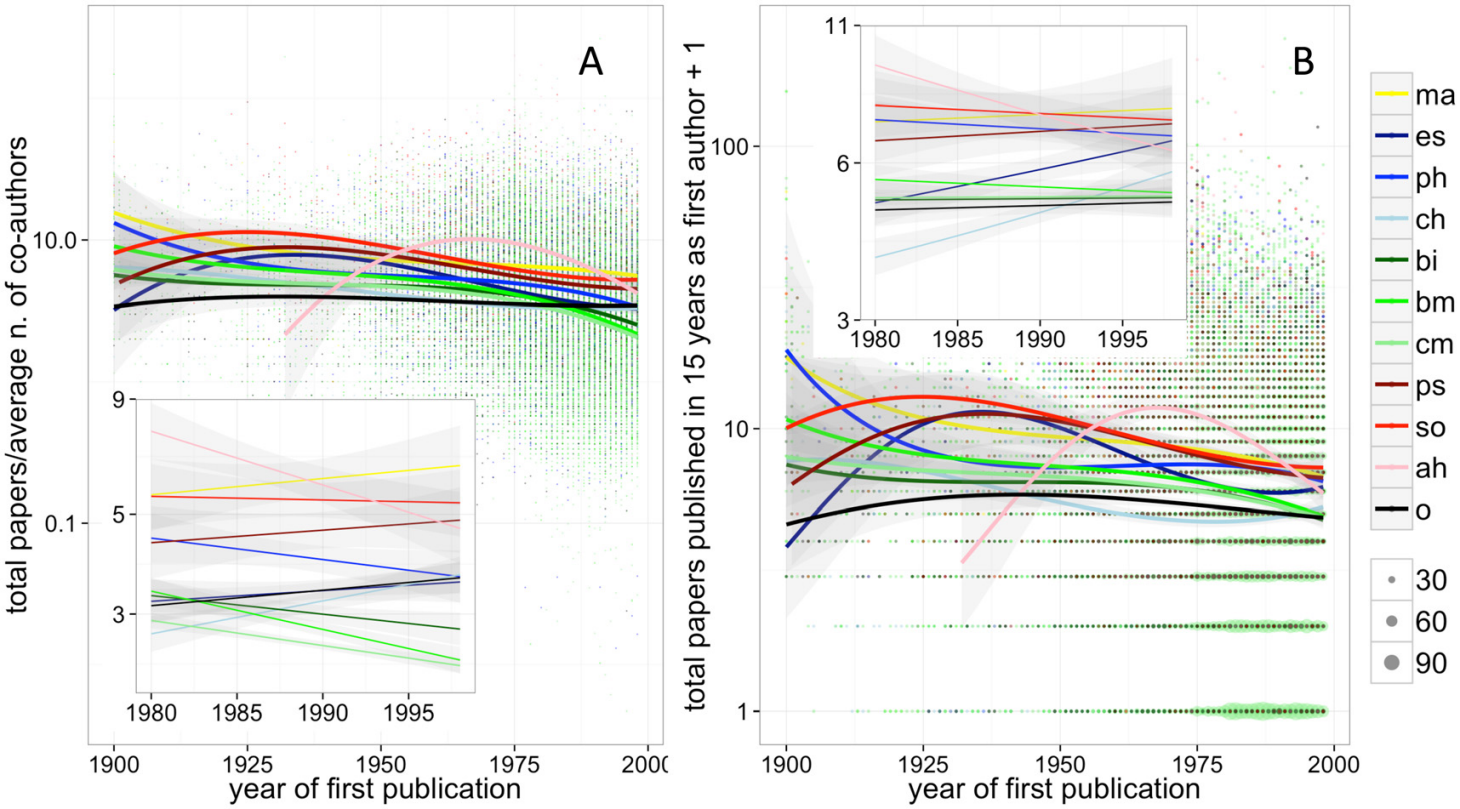
A) Total number of papers published by individual scientists during the first 15 years of publication activity, divided by average number of co-authors per paper, plotted by the year of their first publication. B) Total number of papers published as first author by individual scientists during the first 15 years of publication activity, plotted by the year of their first publication. Boxes in both figures illustrate trends limited to the 1980s. Lines and confidence intervals are derived from a generalized linear model with quasi-Poisson link function (see Methods for further details). Dot size are proportional to data point overlap. Legend: ma = mathematics, es = earth & space science, ph = physics, ch = chemistry, bi = biology, bm = biomedical research, cm = clinical medicine, ps = psychology, so = social sciences, ah = arts & humanities, o = other.

Once publication rates were adjusted for co-authorship, they were no longer increasing. Fractional research productivity, calculated by dividing the total number of papers published by the average number of co-authors, was highest in the first half of the 20^th^ century and declined overall. After the year 1980, fractional productivity has been stable or decreasing for most disciplines, the few exceptions showing modest growth rates (Fig 2a). A multivariable model regressing total productivity on year of first paper and adjusting for co-authorship yielded a substantially similar picture, by suggesting that disciplines underwent, at best, an extremely small increase of fractional productivity (i.e. less than 1% per year) since the 1980s (S1 File).

The number of papers signed as first-author, a position that in most disciplines is bestowed on the team member who mostly contributed to the research [25], had also declined. Researchers who started their careers in 1998 signed as first-authors, on average, two papers less than their colleagues in 1950. Between 1980 and 1998, the number of first-authored papers was non-increasing for all disciplines except Psychology, in which the increase was very modest (less than one extra paper over fifteen years), and Chemistry and Earth and Space Sciences, which started from the lowest rates and have grown rapidly, i.e. from around three to five and four to six first-authored papers, respectively, over 15 years (Fig 2b). If analyses were repeated for the number of (multi-authored) papers published as last author, very similar trends were observed (S1 File).

**Fig 3:**
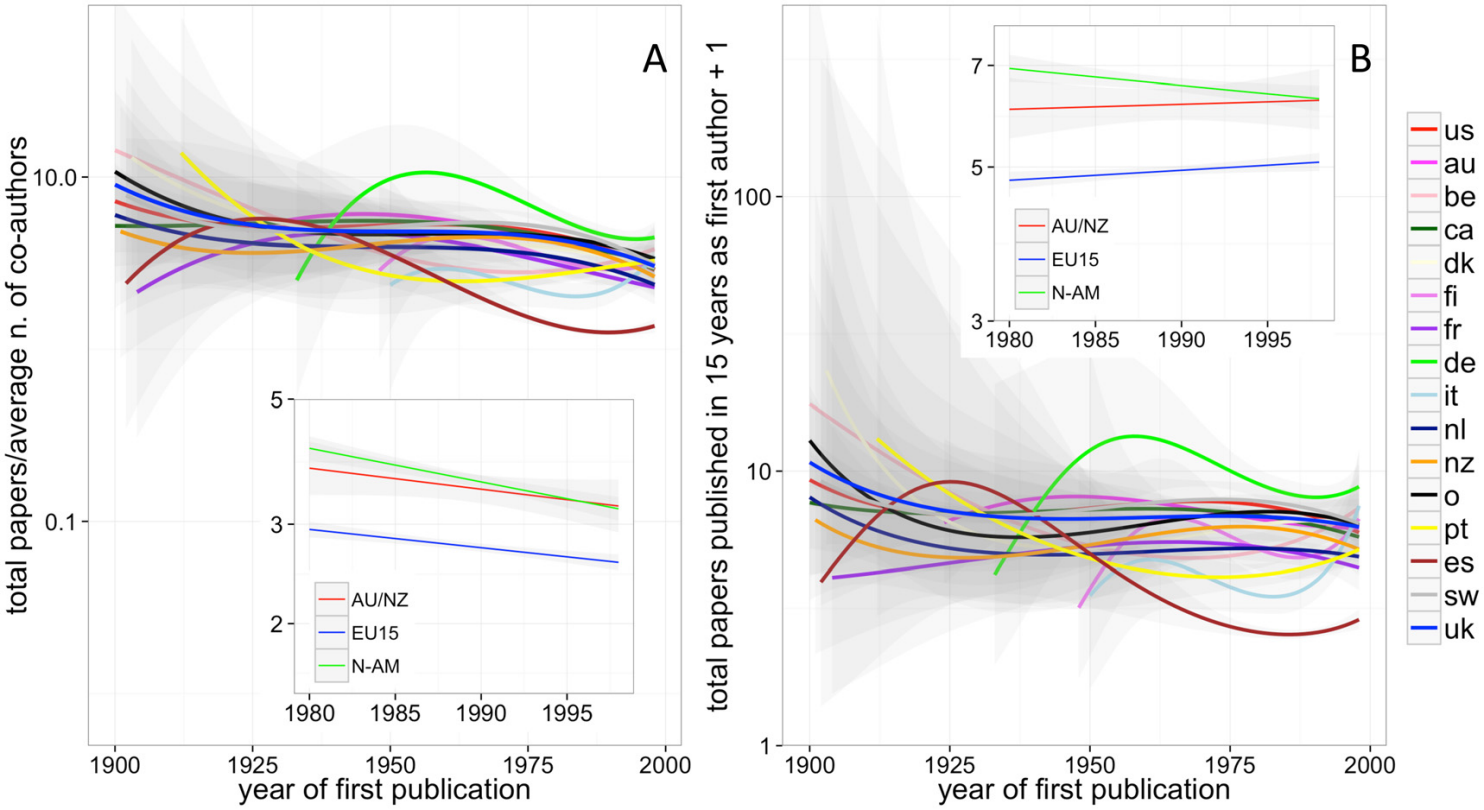
A) Total number of papers published by individual scientists during the first 15 years of publication activity, divided by average number of co-authors per paper, plotted by the year of their first publication, partitioned by geographical area of activity (Australia/New Zealand, Europe-15 Countries, North America). B) Total number of papers published as first author by individual scientists during the first 15 years of publication activity, plotted by the year of their first publication, partitioned by geographical area of activity. Boxes in both figures illustrate trends limited to the 1980s. Lines and confidence intervals are derived from a generalized linear model with Gaussian link function (see Methods for further details). Results for the same data, but partitioned by individual country, are provided in S1 File.

Countries, as captured by our sampling procedure, differed significantly in their average publication and co-authorship rates, but all underwent some increase on both parameters over the years (S1 File). Across geographic regions, fractional and first-author publication rates have followed similar changes over time (Fig 3a and 3b). However, we observed a significant variability between countries. At one extreme were countries that exhibited the highest levels of fractional and first-author publication rates throughout the century, in particular the United States, United Kingdom and Germany. These showed a decrease or no increase (e.g. yearly linear slope±standard error for first authored papers, from 1980-onwards, respectively: -0.004±0.001, -0.000±0.002, -0.002±0.005). At the other extreme are countries that recorded lower publication rates during most of the century and underwent a rapid increase in recent decades. These include Belgium, Portugal, Spain and Italy, for which the rate of first-author publication has tripled since the 1980s (e.g. yearly linear slope for first authored papers, from 1980-onwards, respectively: 0.016±0.007, 0.008±0.004, 0.01±0.003, 0.032±0.008; for numerical results of all countries see S1 File).

Multiple secondary analyses suggested that the patterns observed in this study are genuine, and not artefacts generated by our sampling or analytical strategy. The use of three-initialled names greatly minimized disambiguation errors and, to any extent that such errors affected our sample, they made our analysis conservative with respect to the hypothesis (see Methods). The relative frequency of three-initial names recorded in the Web of Science varied by year and country, but these trends were uncorrelated to the trends observed in our study (S1 Fig, see Methods). Furthermore, names of the 20 most and least productive authors from United States and United Kingdom in our sample did not exhibit any obvious pattern (e.g. an overrepresentation of Asian names) that may suggest the presence of bias in our sample (see Methods, S1 File). The Web of Science database is, like all similar databases, an incomplete representation of the scientific literature, but temporal changes in its coverage are uncorrelated to our findings, and conservative with respect to the hypothesis (S2 Fig, see Methods). We used linear and cubic-polynomial models to illustrate temporal trends, sacrificing detail for the sake of simplicity, but the same fundamental trend (i.e. no increase over time) was observed if a simple mean is calculated year-by-year (S4 Fig). Moreover, we repeated all analyses using alternative “early career” time windows. When the early-career window was set at 25 years (authors’ total N = 21,431), results were very similar to those obtained with a 15-year time window. When the early-career window was limited to the first 8 years of publication activity (N = 51,484), results showed a pronounced decline in individual publication rates (numerical results are reported in S1 File), suggesting that our main results are actually conservative with respect to the hypothesis that early-career researchers are publishing more papers.

## Discussion

We analysed individual publication profiles of over 40,000 scientists whose first recorded paper appeared in the Web of Science database between the years 1900 and 1998, and who published two or more papers within the first fifteen years of activity—an “early-career” phase in which pressures to publish are believed to be high. As expected, the total number of papers published by scientists has increased, particularly in recent decades. However, the average number of collaborators has also increased, and this factor should be taken into account when estimating publication rates. Adjusted for co-authorship, the publication rate of scientists in all disciplines has not increased overall, and has actually mostly declined. Co-authorship might not fairly reflect actual contribution, because authorship attribution practices might have changed over time and therefore roles that previously were not rewarded with authorship—e.g. senior scientist, mentor, lab director, technician, statistician—now might be. However, even if we ignored co-authorship and measured the number of papers published as first author, a position that in most disciplines indicates whom has mostly contributed to the work [25], we observed no significant increase overall. Early-career scientists today publish, as first authors, roughly one paper less than their colleagues in the 1950s.

These results are robust to methodological choices and are conservative with respect to the hypothesis of growing publication rates. Therefore, the widespread belief that pressures to publish are causing the scientific literature to be flooded with salami-sliced, trivial, incomplete, duplicated, plagiarized and false results is likely to be incorrect or at least exaggerated.

If researchers across all disciplines have responded to pressure to publish at all, they might seem to have done so primarily by expanding their network of collaborations within and outside their institution (Fig 1b), thus obtaining (co-)authorship on a higher number of papers with the same amount of research effort. Not all collaborations are alike, and it is possible that specific types of collaboration (e.g. long-distance collaboration versus within-lab collaboration) might have different effects on publication rates. However, it is intuitively clear (and supported by our own data, see S4 Fig) that researchers with multiple collaborators are able to share multiple papers and thereby increase their overall list of publications. Since neither productivity nor impact are typically calculated fractionally by current bibliometric tools, expanding one’s range of collaborations is a virtually cost-free strategy against pressures to publish, and was openly recommended as such in the literature e.g. [10].

There is no denying that co-authorship has grown primarily for genuine scientific necessities linked to the growing complexity of phenomena studied. The fact that co-authorship started growing earlier on in the physical sciences [26], and our finding that, when moving from the humanities to the physical sciences (i.e. from lo-to high-consensus disciplines [24]) the optimal numbers of co-authors increased (S4 Fig) supports this hypothesis. Co-authorship might also have increased thanks to improvements in long-distance communication technology, as well as a growing support for interdisciplinary research. However, the extremely rapid rise in co-authorship observed in biomedical research and other areas suggests that other factors in addition to the growing complexity of science are at play [27,28]. In particular, we hypothesize that performance evaluation policies might represent one of the drivers of increased co-authorship, and therefore that questionable co-authorship practices may be a consequence of pressures to publish that is significantly overlooked by researchers and policy-makers.

The pressures that in numerous surveys and interviews scientists have reported to feel e.g. [16,17] are likely to be genuine. Between-country comparisons made in this study offer a preliminary support of this view. Countries that in our study have higher fractional and first-author productivity, in particular United States and United Kingdom (see S1 File), are also those reporting higher perceived pressures to publish [16, 27]. However, we found no evidence that the output of scientists in these “high-pressure” countries has increased over time, and therefore no indication that scientists in these countries are increasingly fragmenting their output or responding in any other negative way to pressures to publish. This null finding is in agreement with a previous analysis on corrections and retractions, which found no evidence that research integrity might be lower in scientists that publish at higher rates, in high-impact journals or that work in countries where research performance is evaluated quantitatively [29]

The significant increase in fractional and first-author productivity that we observed in South-European countries (i.e. Italy, Spain, Portugal) is likely to reflect not a net increase in productivity, but rather a shift from non-English national journals to English-language international journals indexed in the Web of Science (WOS), a trend largely driven by policies aimed at measuring and maximizing research impact [30]. This trend might be especially pronounced in the social sciences, which traditionally had a national and local focus. Therefore, reports suggesting that journals are flooded with growing submissions e.g. [13, 14] may not be false, but might reflect the growing numbers of researchers in the social sciences and in developing countries who choose to publish in international journals. Between-country comparisons made in this study must, however, be interpreted with caution, because our sampling strategy is probably not random with respect to particular demographics or cultural groups (see Methods). Therefore, future research should confirm our observations about national differences and further examine the link between publication patterns, nationality and research policies.

We found no evidence that our sampling strategy introduced bias in the results, but we cannot exclude that three-lettered names might offer an unbalanced representation of the scientific community. For example, our sampling method could under-represent women, which are more likely than men to change their surname, or it could over-represent Catholics, whom in some countries are more likely to bear three-initialled names. Assessing whether specific cultural groups within a country have different levels of productivity is a fascinating hypothesis to test in future work, but it is unlikely to significantly limit this study’s conclusions, because these are not based on between-country (or between discipline) comparisons but on long-term trends that are similar across countries and disciplines. For our conclusion to be flawed, in other words, one would have to assume that all three-initialled names around the world represent a similar group of people, which are less likely to respond to pressures to publish (or to engage in questionable research practices) than those with two-initialled names—an hypothesis that appears to be rather unrealistic.

Even if, as our data suggests, scientists are not publishing papers at higher individual rates, they might still be experiencing genuine and growing pressures. For example, scientist are likely to experience increasing pressures to write grant applications, reports, syllabi and other material. This would imply that, over time, scientists have been compelled to dedicate a smaller proportion of time to research and publication activities. It is also possible that the average time and effort required by each paper has increased over time, putting successive generations of scientists under growing pressures to maintain a high publication rate. These “increasing workload” and “increasing research effort” hypotheses should be tested in future studies. Nonetheless, these hypotheses would directly contradict the notion, tested in this study, that scientists are increasingly publishing fragmented and inconclusive results, unless one supposed that scientists are publishing papers that are both fewer in number *and* poorer in content.

If, as our data suggests, contemporary science is not suffering from a salami-slicing of papers, then current policies aimed at countering this problem are likely to be ineffective. Indeed, such policies could have negative consequences, because curtailing the list of publications submitted in support of grant applications e.g. [11], or ignoring any consideration of productivity when evaluating institutions’ research performance e.g. [12] might put scientists under even greater pressures to boost their citation scores, journal impact factor profile, visibility in the mass media and to “salami-slice” their collaborations [31], all at the possible expense of scientific quality and rigour.

## Supporting Information

S1 Data Data set underlying all main results reported in the text. (TXT)

S1 Fig Proportion of names with three-initials to all names recorded in the Web of Science each year, for authors listed as first and whose affiliation corresponded to one of the countries included in our analysis (see Materials and Methods for further details). (TIFF)

S2 Fig Simple yearly averages of the parameters reported in Fig 2a and 2b (see Materials and Methods for further details). (TIF)

S3 Fig Proportion of citations going to journals indexed in the Web of Science database, 1900–2013. Lines and confidence intervals are derived from a generalized linear model with quasi-Poisson link function.
(TIF)

S4 Fig Total number of papers (top), average citations (bottom left) and average journal impact factor (bottom right) accrued by individual scientists during the first 15 years of publication activity, plotted by their average number of co-authors. Data was limited to scientists publishing from 1980 onwards. Lines and confidence intervals are derived from a generalized linear model assuming the link function to be quasi-Poisson for total number of papers and Gaussian for average citations and 5-year IF. Legend: ma = mathematics, es = earth & space science, ph = physics, ch = chemistry, bi = biology, bm = biomedical research, cm = clinical medicine, ps = psychology, so = social sciences, ah = arts & humanities, o = other.
(TIFF)

S1 File Printout of results of all analyses underlying the results displayed in figures (i.e. fitted lines) as well as secondary, robustness and sensitivity analyses. (TXT)
