# Supplementary figures and images for: Researchers’ Individual Publication Rate Has Not Increased in a Century
Source: PLoS One. 2016 Mar 9;11(3):e0149504. doi: 10.1371/journal.pone.0149504 (PMC4784736; doi:10.1371/journal.pone.0149504)

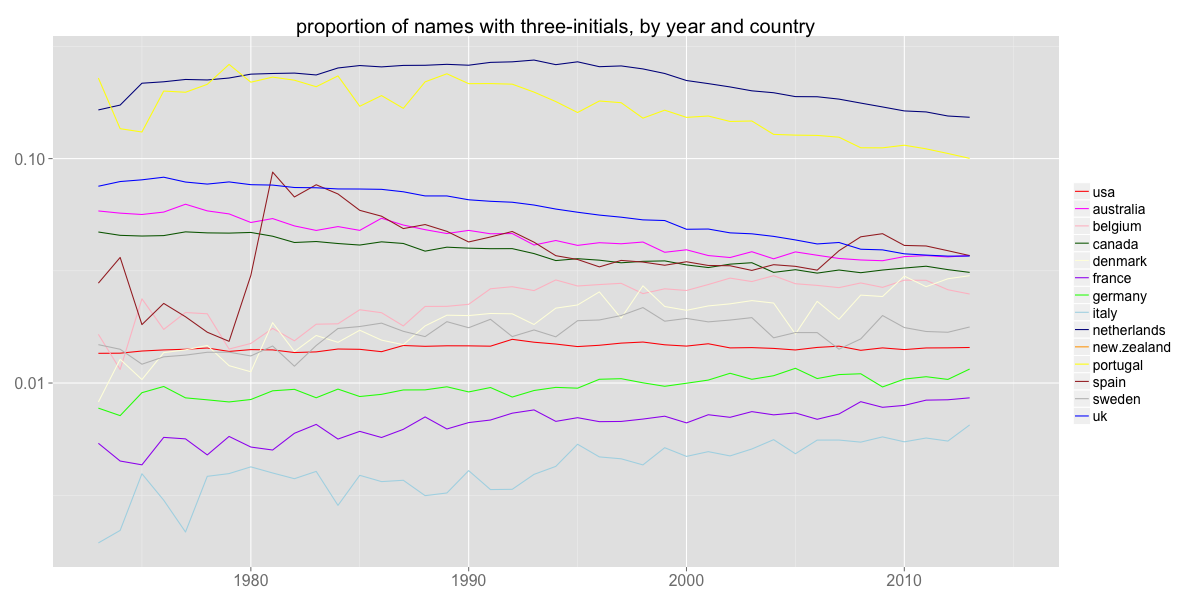

Supplement: S1 Fig — (TIFF) [file pone.0149504.s002.tiff]

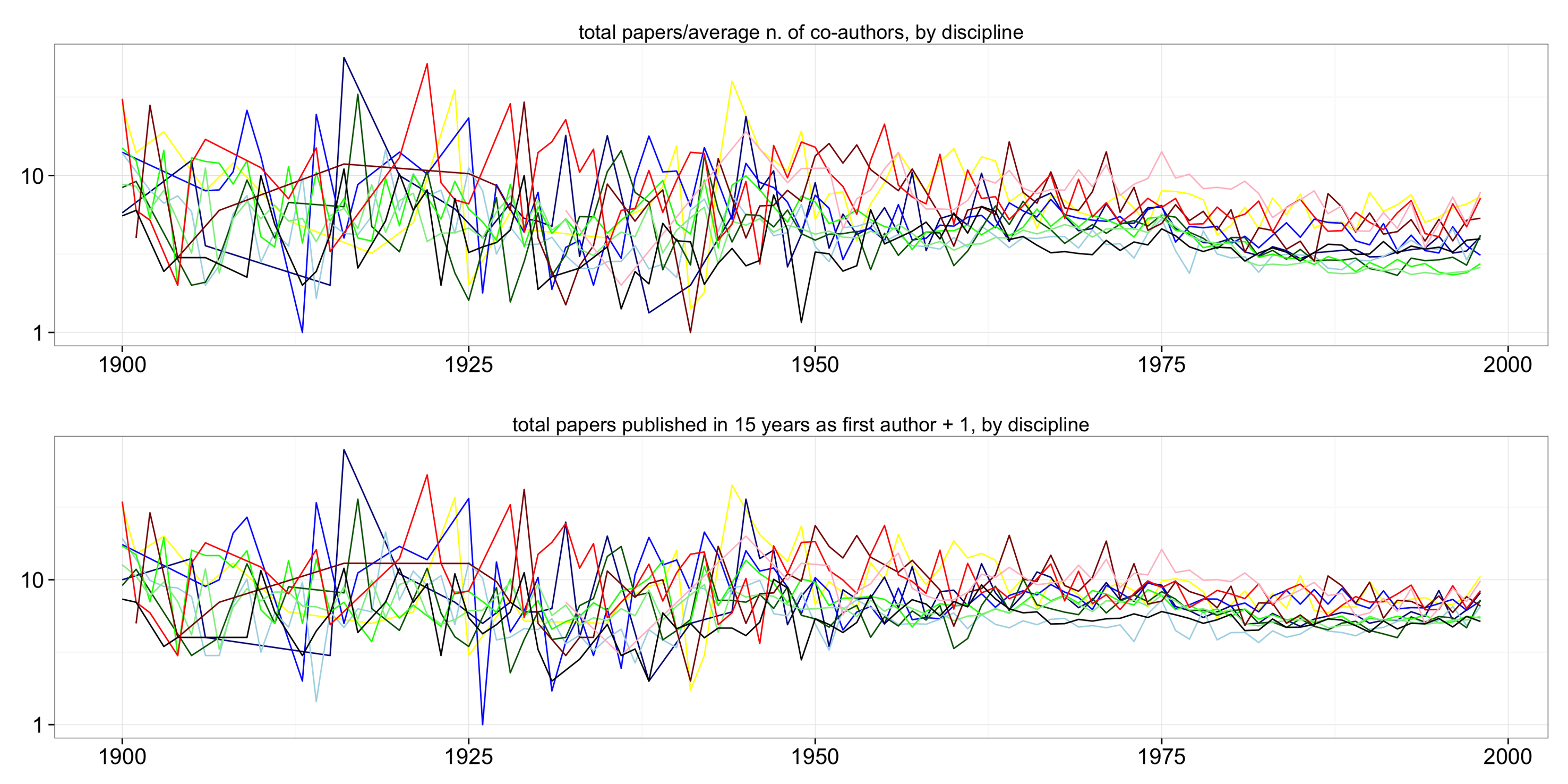

Supplement: S2 Fig — (TIF) [file pone.0149504.s003.tif]

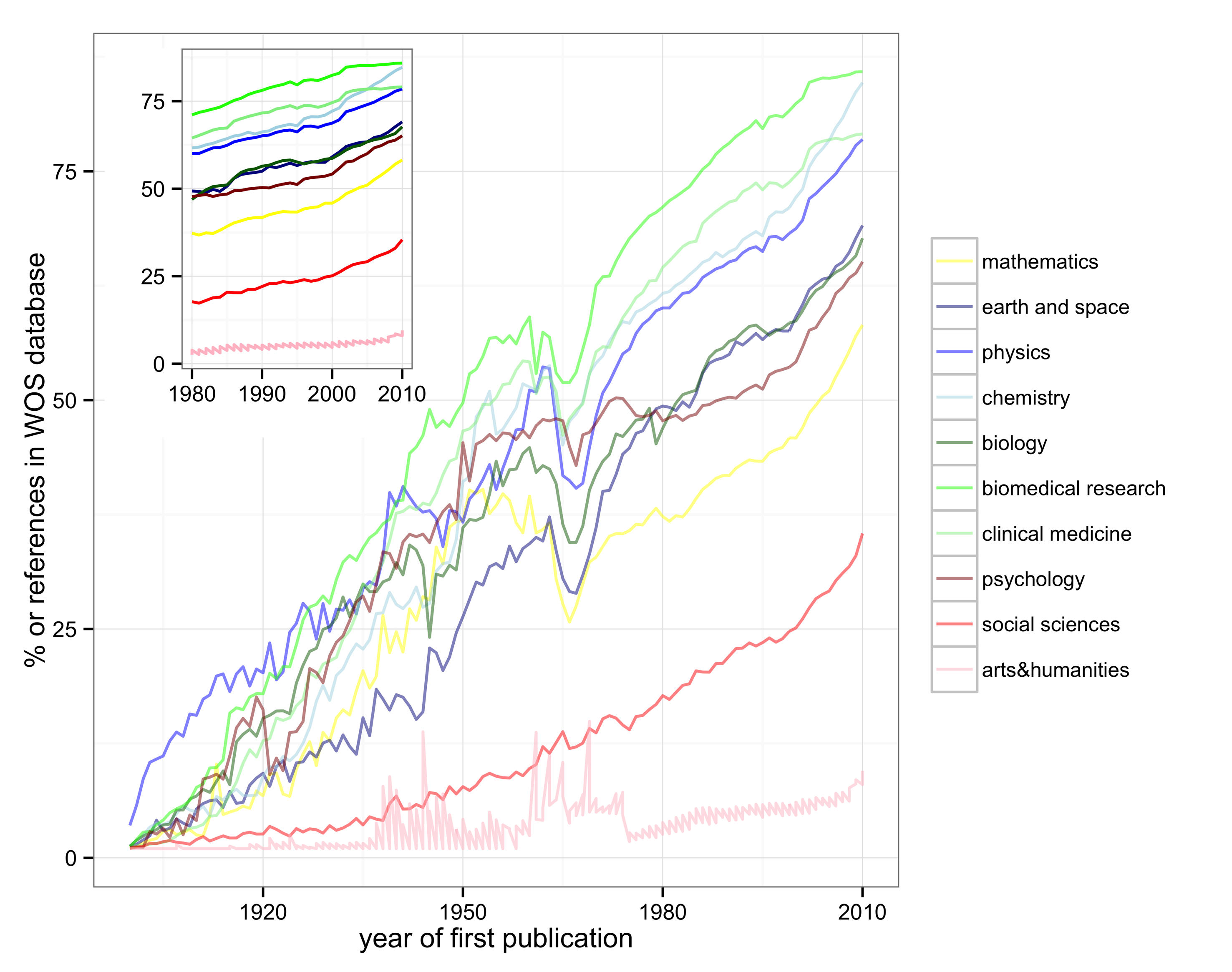

Supplement: S3 Fig — Lines and confidence intervals are derived from a generalized linear model with quasi-Poisson link function. (TIF) [file pone.0149504.s004.tif]

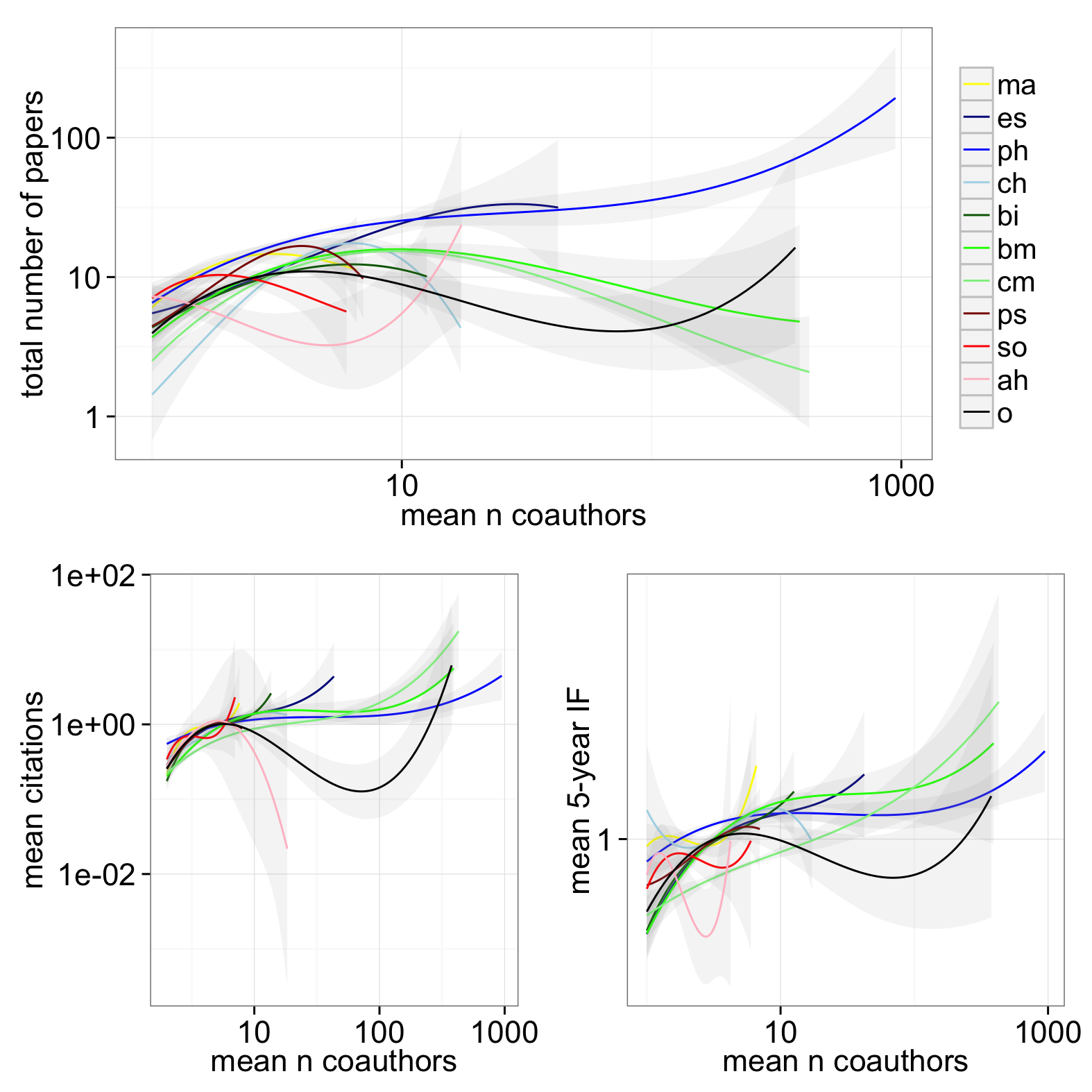

Supplement: S4 Fig — Data was limited to scientists publishing from 1980 onwards. Lines and confidence intervals are derived from a generalized linear model assuming the link function to be quasi-Poisson for total number of papers and Gaussian for average citations and 5-year IF. Legend: ma = mathematics, es = earth & space science, ph = physics, ch = chemistry, bi = biology, bm = biomedical research, cm = clinical medicine, ps = psychology, so = social sciences, ah = arts & humanities, o = other. (TIFF) [file pone.0149504.s005.tiff]
